# Non-invasive estimation of *in vivo* optical properties and hemodynamic parameters of domestic animals: a preliminary study on horses, dogs, and sheep

**DOI:** 10.3389/fvets.2023.1243325

**Published:** 2023-09-18

**Authors:** Lorenzo Frabasile, Caterina Amendola, Mauro Buttafava, Matteo Chincarini, Davide Contini, Bruno Cozzi, Donatella De Zani, Giulia Guerri, Michele Lacerenza, Michela Minero, Lucio Petrizzi, Lina Qiu, Vanessa Rabbogliatti, Emanuela Rossi, Lorenzo Spinelli, Paola Straticò, Giorgio Vignola, Davide Danilo Zani, Emanuela Dalla Costa, Alessandro Torricelli

**Affiliations:** ^1^Dipartimento di Fisica, Politecnico di Milano, Milan, Italy; ^2^PIONIRS s.r.l., Milan, Italy; ^3^Facoltà di Medicina Veterinaria, Università degli Studi di Teramo, Teramo, Italy; ^4^Dipartimento di Biomedicina Comparata e Alimentazione, Università degli Studi di Padova, Legnaro, Italy; ^5^Dipartimento di Medicina Veterinaria e Scienze Animali (DIVAS), Università degli Studi di Milano, Lodi, Italy; ^6^School of Software, South China Normal University, Guangzhou, China; ^7^Istituto Zooprofilattico Sperimentale dell'Abruzzo e del Molise G. Caporale, Teramo, Italy; ^8^Consiglio Nazionale delle Ricerche, Istituto di Fotonica e Nanotecnologie, Milan, Italy

**Keywords:** near infrared spectroscopy, optical properties, differential pathlength factor, hemodynamic properties, domestic animals, skeletal muscle, head, animal welfare

## Abstract

Biosensors applied in veterinary medicine serve as a noninvasive method to determine the health status of animals and, indirectly, their level of welfare. Near infrared spectroscopy (NIRS) has been suggested as a technology with this application. This study presents preliminary *in vivo* time domain NIRS measurements of optical properties (absorption coefficient, reduced scattering coefficient, and differential pathlength factor) and hemodynamic parameters (concentration of oxygenated hemoglobin, deoxygenated hemoglobin, total hemoglobin, and tissue oxygen saturation) of tissue domestic animals, specifically of skeletal muscle (4 dogs and 6 horses) and head (4 dogs and 19 sheep). The results suggest that TD NIRS *in vivo* measurements on domestic animals are feasible, and reveal significant variations in the optical and hemodynamic properties among tissue types and species. In horses the different optical and hemodynamic properties of the measured muscles can be attributed to the presence of a thicker adipose layer over the muscle in the Longissimus Dorsi and in the Gluteus Superficialis as compared to the Triceps Brachii. In dogs the absorption coefficient is higher in the head (temporalis musculature) than in skeletal muscles. The smaller absorption coefficient for the head of the sheep as compared to the head of dogs may suggest that in sheep we are indeed reaching the brain cortex while in dog light penetration can be hindered by the strongly absorbing muscle covering the cranium.

## 1. Introduction

The application of technology and biosensors has been suggested as a method to assess the overall health status of animals. This could allow to evaluate different conditions and monitor the treatment provided in animals that could be associated with the level of animal welfare, with the advantage of being non-invasive (1). Animal welfare is a complex and multi-faceted concept, and it refers to the state of the animal in different conditions including veterinary treatments, animal care and husbandry. Finding non-invasive methods to monitor animal health and welfare parameters is an important goal for both veterinarians and animal welfare scientists (2). In the last decade, technological advances have made available different sensors for monitoring different indicators. Global positioning systems (GPS), accessible data transmission, and accelerometers fitted to collars can give to animal owners the possibility to locate their animals, to track spatio-temporal patterns, and to identify behavioral changes linked to the presence of health issues. Wearable tools for monitoring heart rate or respiratory rate can help monitoring not only health status both in awake and anesthetized animals (3, 4), but also fearful and distressful situations (5). Similarly, remote technologies (e.g., video cameras and imaging systems in the visible and infrared spectral range) can provide insights into animal position and behavior (6), and they can also help the clinician in identifying areas affected by orthopedic diseases (7, 8). Finally, infrared thermography has been studied experimentally and clinically as a support tool to monitor stress and fear (9), surface inflammatory processes or painful conditions (10, 11). However, recent findings highlighted that there is a lack of validation of new technologies in the veterinary field due to the variations between species and breeds and the inadequacy of reference values (12, 13).

Following the latest advances in the biomedical field, near infrared spectroscopy (NIRS) has been proposed as a tool for monitoring hemodynamics in domestic animals, especially in skeletal muscle and cerebral cortex. NIRS is an optical technique that can noninvasively estimate tissue hemodynamics at the microvasculature level by exploiting the different absorption spectra of oxygenated hemoglobin (O_2_Hb) and deoxygenated hemoglobin (HHb) and the ability of near infrared light to penetrate in biological tissue sampling a volume of the order of 1 cm^3^ at a depth of <2 cm. In human applications NIRS is a promising technique, typically applied to noninvasively measure blood volume or total hemoglobin (tHb = O_2_Hb + HHb) and tissue oxygen saturation (S_t_O_2_ = O_2_Hb/tHB) of the cerebral cortex and the skeletal muscle. NIRS can in fact provide biomarker of skeletal muscle fatigue or pathologic conditions like tissue ischemia during muscular rehabilitation or sport exercise (14–16). Moreover, NIRS could offer a simple tool to investigate cortical hemodynamics and functions following motor, cognitive, and somatosensory stimuli or during clinical procedures like general anesthesia, or also during neurorehabilitation (17). In the veterinary field, preliminary NIRS measurements for estimating S_t_O_2_ were reported on dog muscle (18, 19), horse muscle (20–23) and horse head (24). Moreover, NIRS measurements were reported on dog brain (25–27) and on sheep brain (28–33) for monitoring the cortical hemodynamic changes to various external stimuli (e.g., auditory, cognitive). More recently, NIRS was also used to assess physiology in freely moving dolphins and seals (34). Importantly, the overall NIRS data were sometimes contradictory among animal studies, suggesting that NIRS measurement accuracy and reliability need to be improved (33).

In NIRS studies on domestic animals, continuous wave (CW) NIRS commercial devices were used, employing steady state light sources and photodetectors sensitive to light attenuation (18–33). However, in highly diffusive media, like biological tissue in the NIR region, light attenuation is determined by the complex interplay between light absorption (depending on tissue chromophores like hemoglobin, water, lipid, melanin) and light scattering (depending on refractive index changes and tissue structure) (35). Disentangling light absorption (measured by the absorption coefficient μ_a_) from light scattering (measured by the reduced scattering coefficient μ_s_’) is fundamental for obtaining quantitative estimate of the hemodynamic parameters. In CW NIRS this is possible only by adopting a multi-channel tomographic scheme and cumbersome calibration procedures based on tissue phantoms (36). These solutions can be implemented in a research laboratory but are not available in CW NIRS commercial devices that favor easiness, lightness, and portability. Therefore, when using a CW NIRS device (either for human or animal studies) the typical approach is to assume a known value for μ_s_’ or, equivalently, for the differential pathlength factor (DPF) (37). The knowledge of μ_s_’ or DPF enables estimating the hemoglobin concentration changes (ΔO_2_Hb, ΔHHb) with respect to an arbitrary baseline in units of molar concentration (M, *mole/liter*). When the DPF is not known concentration changes are presented in arbitrary units or as the products of concentration changes and mean pathlength, in units of *M cm*. In this latter case the measured parameters are referred to as “pathlength-dependent” hemoglobin concentration. Similarly, by assuming an approximated linear spectral dependance for μ_s_’ and employing a multi-distance CW NIRS approach (typically named space-resolved spectroscopy, SRS), it is possible to estimate S_t_O_2_ (38). When performing CW NIRS data analysis, mean values for μ_s_’ or DPF are usually taken from literature. However, to the best of our knowledge, consistent data on tissue optical properties of human tissue are limited (34), and even less data are available for domestic animals. Few *ex vivo* or *post mortem* data on domestic animal optical properties are in fact reported (39–41), but it is well known that the procedures for *ex vivo* tissue treatment (e.g., fixation, staining) and the natural physiological phenomena occurring *post mortem* (e.g., *rigor mortis*) can significantly alter the optical properties as compared to the *in vivo* situation (42).

**Figure 1:**
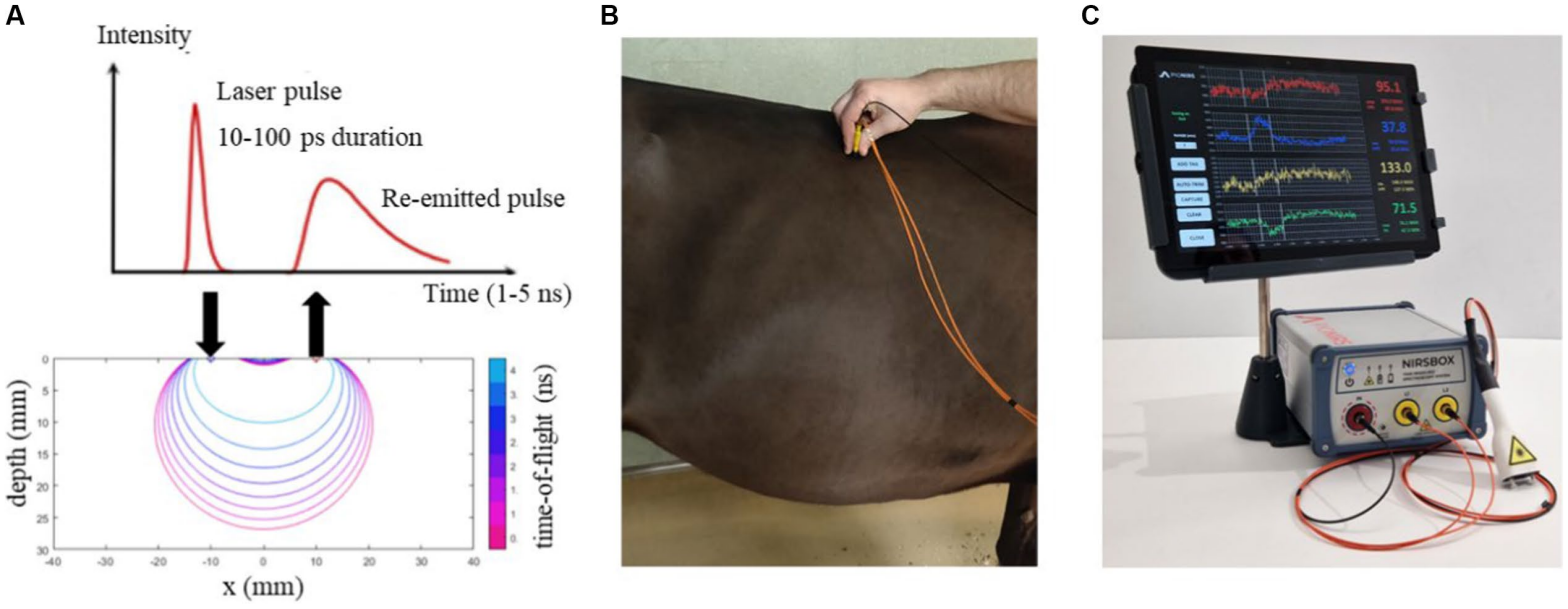
**(A)** Scheme of TD NIRS measurement and of sensitivity maps as a function of photon time-of-flight; **(B)** Photo of TD NIRS measurement on a horse; **(C)** Photo of the NIRSBOX device with the dedicated probe.

From the above observations, the lack of consistent *in vivo* data for the optical properties of domestic animals becomes evident: using wrong values for μ_s_’ or DPF may jeopardize the interpretation of CW NIRS data acquired. The aim of this work is to provide *in vivo* measurement of the tissue optical properties (μ_a_, μ_s_’, and DPF) and of the hemodynamic parameters (O_2_Hb, HHb, tHb and S_t_O_2_) of different domestic animal species. Specifically, we focused on the skeletal muscle in dog and horse and of the head of dog and sheep. The choice of the species was dictated by their great importance in the veterinary field. Moreover, state-of-the-art time domain (TD) NIRS systems and a physical model for photon diffusion were used. TD NIRS is a particular implementation of NIRS that uses short (picosecond) laser pulses and fast optoelectronic devices for measuring the photon distribution of time of flight in the tissue (see Figure 1A) from which optical properties can be estimated (43). In the past decades, TD NIRS was more complex, expensive, and bulky than CW NIRS, therefore its use was limited mostly to academic or research laboratories. Nowadays, thanks to the incessant development of optoelectronics, also TD NIRS has significantly reduced dimensions and complexity allowing for a simpler use, lightness, and portability and enabling measurements out of the research laboratories (44).

## 2. Materials and methods

### 2.1. Ethical statement

All measurements on animals were carried out in accordance with relevant guidelines and regulations, and all experimental protocols were reviewed and approved by the Italian Health Ministry according to Directive 2010/63/EU (n. 457/2016-PR, 919/2017-PR; n. 0022387/2020). Verbal informed consent was gained from each horse and dog owner prior to taking part in this research, written consent was deemed unnecessary as no personal details of the participants were recorded. If any animal was deemed to be in greater than mild stress (assessed live by an independent veterinarian), then it would immediately be excluded from the study.

### 2.2. Animals

#### 2.2.1. Horses

Six horses with different coat color were recruited, their characteristics (age, gender, coat color, and breed) are reported in Table 1. All the animals were healthy and did not undergo any treatments before the measurements. During the measurements, an operator restrained the animal with a head collar and lead rope, while a veterinarian gently applied the probe to the horse’s body areas of interest. The measurements were taken on both sides of the horses on three different areas, corresponding to *Triceps Brachii*, *Gluteus Superficialis*, and *Longissimus Dorsi*. Each position was measured 10 times, and each repetition had an integration time of 0.5 s. Measurements were performed with the NIRSBOX TD NIRS system (see Section 2.3.1). The distance between the injection and detection fiber was 3 cm. The measurements were carried out during the summer season, and the horse’s hair never exceeded 3 cm in length. Figure 1B shows an example of TD NIRS measurement on a horse.

**Table 1:** The characteristics (age, sex, coat color, and breed) of the measured horses.

Horse [#]	Age [years]	Sex	Coat color	Breed
1	7	MC	Gray	Paint Horse
2	8	M	Black	Paint Horse
3	17	MC	Bay	Half-breed
4	13	F	Chestnut	Poland
5	26	MC	Chestnut Tobiano	Paint Horse
6	16	M	Chestnut	Haflinger

M, male; MC, castrated male; F, female.

#### 2.2.2. Dogs

Four dogs with long- and short-hair were recruited and measured. Table 2 reports their characteristics (age, gender, coat color, and breed). All the animals were in good physical condition (body condition score equal to 3/5). No shaving was required, guaranteeing a fully non-invasive process. Measurements were taken with the dog gently restrained by their owner on *Triceps Brachii*, *Biceps Femoralis*, and on Forehead (far from sinus cavities, at the level of temporalis musculature). To ensure the repeatability of the measurements, a veterinarian gently applied the probe on each site three times by replacing the probe on the tissue, and the procedure was replicated on both sides of the animal. Each measurement consisted of 10 repetitions, with an integration time of 0.5 s for each repetition, while maintaining a fixed distance of 2.5 cm between the injection and detection fibers. Measurements were performed with the NIRSBOX TD NIRS system (see Section 2.3.1).

**Table 2:** The characteristics (age, sex, coat color, and breed) of the measured dogs.

Dog [#]	Age [years]	Sex	Coat color	Breed
1	4	F	Light gray	Skye Terrier
2	13	M	Black and tan	German Shepherd
3	8	M	Black and white	Mixed breed
4	7	F	Brown and white	Mixed breed

M, male; F, female.

#### 2.2.3. Sheep

Nineteen Sarda sheep of different age were selected from the same flock (Table 3). All sheep were no gestating nor lactating. Measurements were performed with the multiwavelength TD NIRS system (see Section 2.3.2) with a source detector distance ρ = 2.5 cm over the right and left hemisphere while they were standing still, gently restrained by two operators. Shaving of the head was manually performed the day before the measurements to remove the excess wool. The acquisition time was 1 s for each wavelength, while <10 s were needed to change wavelength and optimize the collected signal. Five repetitions for each position were acquired, resulting overall in about 4 min for each sheep.

**Table 3:** The characteristics (age, sex, coat color, and breed) of the measured sheep.

Sheep [#]	Age [years]	Sex	Coat color	Breed
1–9	0.7–1	F	White	Sarda
10–12	1–2	F	White	Sarda
13–15	3–4	F	White	Sarda
16–19	6–7	F	White	Sarda

F, female.

### 2.3. TD NIRS systems

#### 2.3.1. NIRSBOX device

The NIRSBOX device (PIONIRS Srl. Italy) was used to noninvasively measure skeletal muscle and head in dogs and the skeletal muscle in horses. The NIRSBOX device is a single channel TD-NIRS device. It is characterized by limited weight, portability, and compactness. It has two pulsed laser diodes at 685 nm and 830 nm, and a time-to-digital-converter and timing electronics with a temporal resolution of 10 ps for recording of the photon distribution of time-of-flight (DTOF). Laser light is injected and collected from the tissue through an optical custom probe. The probe was designed to be ergonomic and easy to use. The probe is characterized by two teeth which allowed the light to penetrate inside the hairs of the animal and 90 degrees bending optical interface (see Figure 1C). The probe is linked to the device by a 2-m long fiber bundle composed by two fibers (0.1 mm core, multimode graded index, silica) on the injection side and a 2-m long collection fiber (1 mm core, multimode, graded index, POF). Inside the tissue the photons are backscattered and sent to the photodetector (Silicon PhotoMultiplier, SiPM, with an active area of 1.7 mm^2^). The measured Instrument Response Function (IRF), obtained by facing the injection fiber and collection fiber, has a full-width-at-half-maximum around 200 ps. More details on the NIRSBOX device can be found in Lacerenza et al. (45).

#### 2.3.2. Multi-wavelength laboratory device

A multiwavelength TD-NIRS system was used to estimate the optical properties and the DPF of the head of the sheep. A supercontinuum fiber laser (SC450-6 W, Fianium, UK) and a set of interference filters (Hard Coated OD 4 10 nm Bandpass Filters, Edmund Optics Ltd. UK) were used to sequentially produce laser pulses (duration <100 ps, repetition rate 37 MHz, average power <2 mW) at 670 and 830 nm. Multimode graded index glass (core diameter 0.1 mm) and plastic (core diameter 1.0 mm) optical fibers were used to inject and collect light into the head of the sheep, respectively. A home-made solid-state large area SiPM detector (developed at the Department of Physics of Politecnico di Milano) and a time-correlated single photon counting board (SPC130 Becker-Hickl GmbH, Germany) were used to acquire the photon DTOF. More details on the multi-wavelength TD NIRS device can be found in Zhao et al. (46).

### 2.4. TD NIRS data analysis

**Figure 2:**
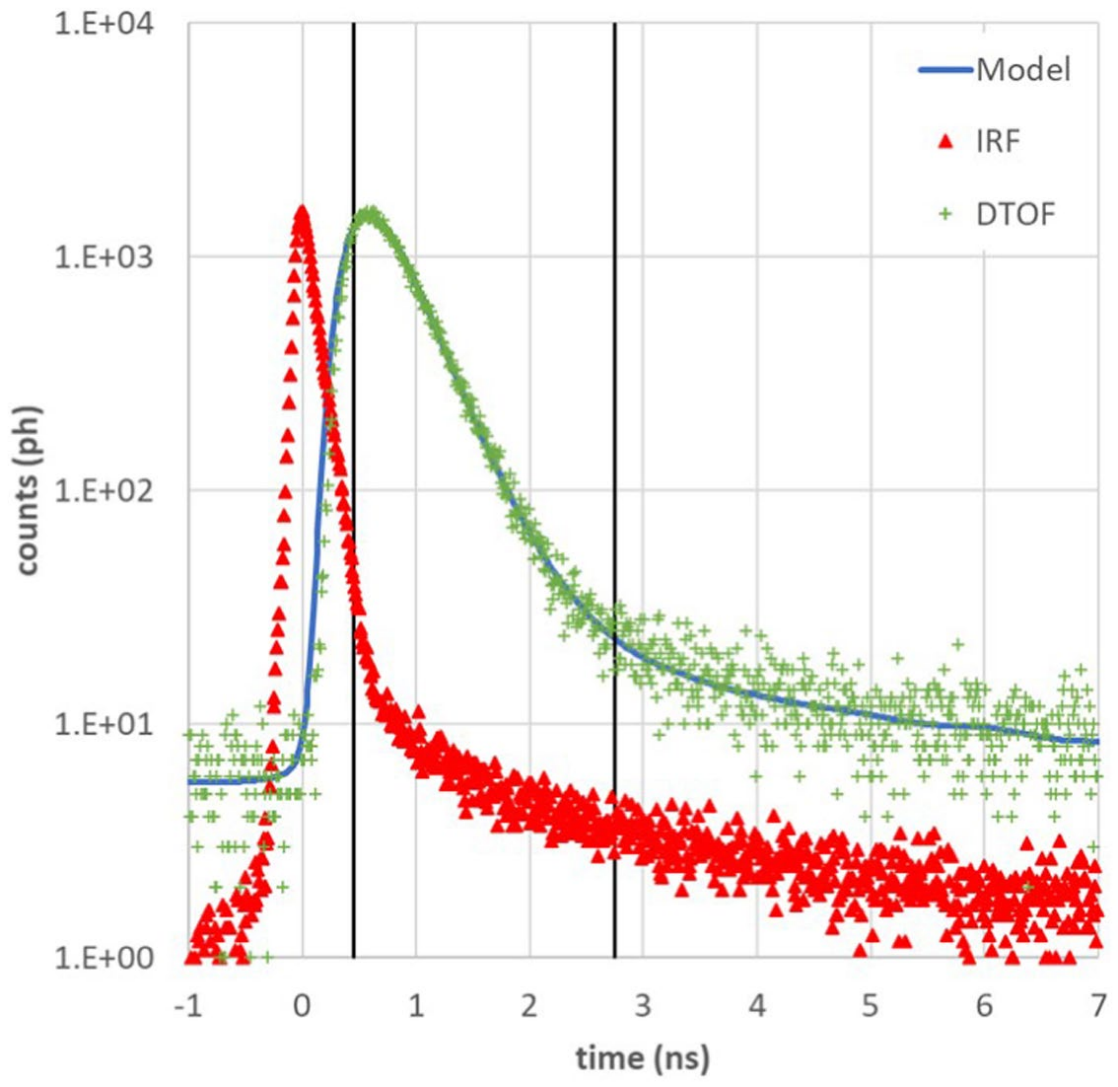
Example of photon distribution of time of flight (DTOF, green +) at 685 nm acquired on *Biceps Femoralis* of a dog, instrument response function (IRF, red ∆), and theoretical model (blue line). Black vertical lines represent the fitting range from 80% on the left to 1% on the right of the DTOF peak.

A physical model for time-resolved reflectance in a homogeneous medium (47) was used to fit the DTOF after convolution with the IRF (48). The fitting range was fixed at 80 and 1% of the peak on the leading and trailing edge of the DTOF. In the fitting procedure, μ_a_ and μ_s_’ were considered as free parameters, while source-detector distance ρ and the time origin *t*_0_ (set by the barycenter of the IRF) were kept as fixed values. A constant background noise was subtracted from each measured DTOF to remove dark counts in the photodetector and ambient light noise. From the DTOF we also estimated the DPF as DPF = *v* < *t* > /ρ, where *v* = *c*/*n* is the speed of light, *n* = 1.4 is the refractive index, and < *t* > is the photon mean time-of-flight (i.e., the barycenter or first order moment of the DTOF) (37, 49, 50). An example of DTOF, IRF and fit is reported in Figure 2. We have then fitted the μ_a_ data at 670 nm and 830 nm to a linear combination of the contributions from HHb and O_2_Hb by means of the Beer’s law using the specific absorption of hemoglobin in dog, horse, and sheep (51). From HHb and O_2_Hb, then tHb and S_t_O_2_ were calculated.

### 2.5. Statistical analysis

The statistical analysis was performed using IBM SPSS Statistics 28 (SPSS Inc., Chicago, United States). Descriptive statistics were carried out for each animal species and different tissue types, data were presented as mean and standard deviation (mean ± SD). As the sample size was small, non-parametric test were used. Kruskal–Wallis test was conducted to investigate possible differences in the optical properties and the hemodynamic parameters for the considered animal species and different tissue types, *post hoc* comparisons were conducted using Mann–Whitney Tests with a Bonferroni adjusted *p* value for multiple tests. Differences were considered to be statistically significant if *p* ≤ 0.05.

## 3. Results

Table 4 reports the optical properties (absorption coefficient μ_a_ [cm^−1^], reduced scattering coefficient μ_s_’ [cm^−1^], and differential pathlength factor *DPF* [−]) at 670 nm or 685 nm and at 830 nm, and the hemodynamic parameters (deoxygenated hemoglobin, HHb [μM]; oxygenated hemoglobin O_2_Hb [μM]; total hemoglobin, tHb [μM]; tissue oxygen saturation, S_t_O_2_ [%]) for the different tissue types. Values are averages and corresponding standard deviations across all animals, all repetitions, and locations (i.e., left side and right side).

**Table 4:** The optical properties (absorption coefficient μ_a_ [cm^−1^], reduced scattering coefficient μ_s_’ [cm^−1^], and differential pathlength factor DPF [−]) and the hemodynamic parameters (deoxygenated hemoglobin, HHb [μM]; oxygenated hemoglobin O_2_Hb [μM]; total hemoglobin, tHb [μM]; tissue oxygen saturation, S_t_O_2_ [%]) for the different tissue types.

		μ_a_ [cm^−1^]		μ_s_’ [cm^−1^]		DPF [−]		HHb [μM]	O_2_Hb [μM]	tHb [μM]	S_t_O_2_ [%]
Species	Tissue	RED	IR	RED	IR	RED	IR				
Horse	*Triceps Brachii*	0.48 ± 0.11	0.31 ± 0.06	8.5 ± 1.3	5.1 ± 0.5	3.4 ± 0.3	3.2 ± 0.3	62.1 ± 15.7	75.3 ± 20.6	137.4 ± 29.0	54.7 ± 7.1
	*Longissimus Dorsi*	0.17 ± 0.09	0.16 ± 0.06	11.0 ± 1.4	8.7 ± 0.9	6.5 ± 1.1	6.2 ± 1.3	20.7 ± 11.6	40.3 ± 18.6	61.0 ± 29.4	67.3 ± 5.2
	*Gluteus Superficialis*	0.20 ± 0.14	0.17 ± 0.10	11.1 ± 2.2	8.7 ± 1.6	6.3 ± 1.5	6.3 ± 1.7	25.0 ± 16.9	42.8 ± 30.4	67.8 ± 46.6	63.6 ± 4.3
Dog	*Triceps Brachii*	0.24 ± 0.12	0.19 ± 0.07	12.5 ± 3.0	7.8 ± 1.9	5.9 ± 1.2	5.1 ± 1.1	41.3 ± 22.6	40.3 ± 12.0	81.6 ± 34.3	51.6 ± 6.4
	*Biceps Femoralis*	0.18 ± 0.07	0.16 ± 0.05	12.1 ± 1.8	8.1 ± 1.5	6.4 ± 0.9	5.7 ± 1.0	30.8 ± 11.6	34.9 ± 14.7	65.6 ± 25.8	52.8 ± 4.2
	Head	0.42 ± 0.13	0.33 ± 0.08	14.1 ± 4.8	6.6 ± 2.6	4.7 ± 0.7	3.5 ± 0.9	72.8 ± 23.8	76.7 ± 20.3	149.5 ± 40.9	51.6 ± 5.2
Sheep	Head (0.67 < year < 1)	0.24 ± 0.03	0.17 ± 0.02	13.8 ± 1.8	10.6 ± 1.6	6.0 ± 0.5	6.2 ± 0.5	25.3 ± 3.8	51.8 ± 7.0	77.1 ± 9.0	67.1 ± 3.6
	Head (1 < year < 2)	0.19 ± 0.02	0.14 ± 0.01	13.2 ± 2.3	10.8 ± 1.8	6.4 ± 0.5	6.8 ± 0.5	19.3 ± 2.5	44.8 ± 5.8	64.1 ± 5.6	69.7 ± 4.3
	Head (3 < year < 4)	0.22 ± 0.02	0.16 ± 0.01	15.5 ± 2.3	12.0 ± 1.8	6.7 ± 0.7	6.9 ± 0.6	22.2 ± 3.0	48.6 ± 2.2	70.8 ± 3.7	68.8 ± 3.1
	Head (6 < year < 7)	0.17 ± 0.03	0.12 ± 0.02	8.8 ± 1.0	7.2 ± 0.9	5.5 ± 0.5	5.9 ± 0.4	17.4 ± 3.5	35.9 ± 6.1	53.3 ± 9.0	67.3 ± 3.1

Values are average and corresponding standard deviation across all repetitions and locations. RED = 685 nm for horse and dog, 670 nm for sheep. IR = 830 nm.

In the measured horses, both optical properties and the hemodynamic parameters differed in the three muscles considered (Kruskal–Wallis test, *p* < 0.001). The *Triceps Brachii* muscle presents significantly higher μ_a_ (0.48 ± 0.11 cm^−1^ at 685 nm and 0.31 ± 0.06 cm^−1^ at 830 nm) as compared to the *Longissimus Dorsi* (0.17 ± 0.09 cm^−1^ at 670 nm and 0.16 ± 0.06 cm^−1^ at 830 nm; Mann–Whitney test, *p* = 0.000 and *p* = 0.000, respectively) and to the *Gluteus Superficialis* (0.20 ± 0.20 cm^−1^ at 685 nm and 0.17 ± 0.10 cm^−1^ at 830 nm; Mann–Whitney test, *p* = 0.033 and *p* = 0.030, respectively). Besides, the *Gluteus Superficialis* presents significantly higher μ_a_ at 685 nm and at 830 nm compared to the *Longissimus Dorsi* (Mann–Whitney test, *p* = 0.033 and *p* = 0.033, respectively). Moreover, the *Triceps Brachii* shows a significantly lower μ_s_’ (8.5 ± 1.3 cm^−1^ at 685 nm and 5.1 ± 0.5 cm^−1^ at 830 nm) than *Longissimus Dorsi* (11.0 ± 1.4 cm^−1^ at 685 nm and 8.7 ± 0.9 cm^−1^ at 830 nm; Mann–Whitney test, *p* = 0.000 and *p* = 0.000, respectively) and *Gluteus Superficialis* (11.1 ± 2.2 cm^−1^ at 685 nm and 8.7 ± 1.6 cm^−1^ at 830 nm; Mann–Whitney test, *p* = 0.001 and *p* = 0.008 respectively). No differences were found in the μ_s_’ between the *Longissimus Dorsi* and the *Gluteus Superficialis*. The observed differences in μ_a_ and μ_s_’ between *Triceps Brachii* and the other horse muscles are reflected also in the DPF values. The DPF value in the *Triceps Brachii* (3.4 ± 0.3 at 685 nm and 3.2 ± 0.3 at 830 nm) is in fact almost half of the values of the *Longissimus Dorsi* (6.5 ± 1.1 at 685 nm and 6.2 ± 1.3 at 830 nm) and of the *Gluteus Superficialis* (6.3 ± 1.5 at 685 nm and 6.3 ± 1.7 at 830 nm).

In the measured dogs, the optical properties of the two types of skeletal muscles are different and so are the corresponding hemodynamic parameters. The *Triceps Brachii* shows a larger μ_a_ (0.24 ± 0.12 cm^−1^ at 685 nm and 0.19 ± 0.07 cm^−1^ at 830 nm) than the *Biceps Femoralis* (0.18 ± 0.07 cm^−1^ at 685 nm and 0.16 ± 0.05 cm^−1^ at 830 nm, Mann–Whitney test, *p* < 0.001 and *p* < 0.001 respectively), and this is also reflected in a larger tHb (81.6 ± 34.3 μM in the *Triceps Brachii*, 65.6 ± 25.8 μM in the *Biceps Femoralis*). The μ_s_’ are significantly different in the two muscles (Mann–Whitney test, *p* < 0.001). Noticeably, the head presents much higher absorption values as compared to the skeletal muscles (0.42 ± 0.13 cm^−1^ at 685 nm and 0.33 ± 0.08 cm^−1^ at 830 nm, Kruskal–Wallis test, p < 0.001; Mann–Whitney test; *p* = 0.000 and *p* = 0.030 compared to *Biceps Femoralis* and *Triceps Brachii* respectively), resulting in lower DPF (4.7 ± 0.7 at 685 nm and 3.5 ± 0.9 at 830 nm) than in the muscles, and in hemodynamic parameters with larger tHb (149.5 ± 40.9 μM) and comparable S_t_O_2_ with respect to the muscles.

In the measured sheep, the absorption coefficient of the head shows a significant decrease with age (Kruskal–Wallis test, *p* < 0.001), and a corresponding reduction of the hemodynamic parameter with a minimum value of tHb equal to 53.3 ± 9.0 μM. It can be observed that these values are much lower than those obtained and on head of dogs.

The oldest sheep (6–7 years old) have a significantly lower scattering coefficient (8.8 ± 1.0 cm^−1^ at 670 nm and 7.2 ± 0.9 cm^−1^ at 830 nm) compared to the other age groups (minimum values of 13.2 ± 2.3 cm^−1^ at 670 nm for age group 1–2 years, and 10.6 ± 1.8 cm^−1^ at 830 nm for age group <1 year; Kruskal–Wallis test, p < 0.001). Similarly, the DPF is lower in oldest animals (5.5 ± 0.5 at 670 nm and 5.9 ± 0.4 at 830 nm).

## 4. Discussion

### 4.1. Novelty of this work

We presented the assessment of *in vivo* optical properties and hemodynamic parameters of different tissues (skeletal muscle, head) of different domestic species (horse, dog, sheep) by TD NIRS measurements. The few data reported in the literature refers to *ex vivo* or *post-mortem* specimens where optical parameters and hemodynamic parameters can significantly change with respect to the *in vivo* case due to sample fixation procedures and normal physiological events occurring in the specimens. Therefore, the data presented in this work, although preliminary since sample size was determined by the availability of healthy animals, represent a significant contribution.

Another important result is related to the feasibility of *in vivo* TD NIRS measurements on domestic animals. During TD NIRS measurement sessions no animal showed discomfort or stress (as evaluated by the experienced veterinarian participating to the study without the use of a scale) and no animal was excluded from the study. In most cases the signal-to-noise- ratio (SNR) was adequate, the hair created a minimal SNR reduction that was mitigated by using a custom probe. No shaving procedures were adopted in horses and dogs while for sheep shaving the head did not cause any issue since it is a normal procedure for this species. Skin color can indeed be the main cause of limited SNR, especially in horses with dark black skin pigmentation (41). However, in dogs and sheep no issues were found.

### 4.2. Interpretation of results

The optical properties of animal tissues are comparable to those of human tissues in the same spectral range and similarly all animal tissues show a slight decrease with wavelength for both μ_a_ and μ_s_’ (52). For the absorption coefficient the decrease can be explained considering the intrinsic absorption spectra of HHb and O_2_Hb, the main chromophores in the wavelength range considered (51). As for the reduced scattering coefficient, it is the physics of the interaction between light at different wavelengths and scattering particles that plays a main role. These results support the use of NIRS also on animals, even if NIRS devices are not specifically designed or optimized for use with animals, apart few exceptions (33, 34, 53).

In horses the difference between optical properties of the measured muscles can be attributed to the presence of a thicker adipose layer over the muscle in the *Longissimus Dorsi* and in the *Gluteus Superficialis* as compared to the *Triceps Brachii*. Fat tissue has in fact a lower μ_a_ and higher μ_s_’ as compared to muscle tissue (54), therefore the maximum photon penetration depth in the tissue is reduced (55). The measurement performed on the *Triceps Brachii* preferentially samples the muscle bundles resulting in high μ_a_ and low μ_s_’ values, while in the *Gluteus Superficialis* and the *Longissimus Dorsi* the measurement is much more affected by the optical properties of the overlying fat resulting in a lower μ_a_ and higher μ_s_’ values. The hemodynamic parameters support the aforementioned statements. In particular, the higher hemoglobin concentrations in the *Triceps Brachii* muscle (tHb = 137.4 ± 29.0 μM) compared to the *Longissimus Dorsi* (tHb = 61.0 ± 29.4 μM) and to the *Gluteus Superficialis* (tHb = 67.8 ± 46.6 μM) reflect the presence of superficial adipose layers in these muscles. Finally, we note that the dispersion of the data (as gaged by the standard deviation over repetitions and positions) for all parameters is larger for the *Gluteus Superficialis* than for the other muscles. This difference is probably related to the fact that the *Gluteus Superficialis* is one of the largest muscles in the horse and can vary significantly across horses depending on training intensity, free exercise, or posture (56).

In dogs the absorption coefficient is higher in the temporalis musculature than in skeletal muscles. This is probably because the dog head tissue is composed by a layered structure with skin and by the thick temporal muscle, essential for opening and closing the mouth and for chewing. The other species also possess the same muscle, but rhythmical chewing of herbivores and omnivores requires the development of the masseter muscle more than the temporal (57). The probe was in fact positioned over the temporal region, far from sinus cavities. The reported values for the head are therefore an average of all these tissues, where the muscle has a greater contribution. This is also supported by the fact that μ_a_ in the dog head is similar to μ_a_ in horse *Triceps Brachii*. The larger μ_s_’ in dog head as compared to *Triceps Brachii* in horse is due to the presence of the skull with bone having a higher μ_s_’ than soft tissues (39).

In all dog tissues the values for S_t_O_2_ are slightly larger than 50% and therefore seem a bit lower than expected. Indeed, NIRS signals reflects the perfusion at the microcirculation level, and we expect a larger contribution from venous than arterial blood. However, like in human measurements, expected data for S_t_O_2_ should be closer to 60% or 75% rather than 50%. Possible causes of this can be the fact that we have neglected the contribution of other tissue chromophores (e.g., water) (58), the use of values for the specific absorption of hemoglobin that are not optimal (59), or also the effect of heterogeneity in the tissue (modeled as homogeneous infinite medium) (60).

The smaller absorption coefficient for the head of the sheep as compared to the head of dogs may suggest that in sheep we are indeed reaching the brain cortex as also demonstrated by other NIRS studies (33) while in dog light penetration is hindered by the strongly absorbing muscle covering the cranium. The decrease in the reduced scattering coefficient for the oldest sheep can be related to changes in the thickness of the skull and the relative progressive enlargement of the frontal respiratory sinuses induced by aging. We note that in sheep head there is a significant reduction of O_2_Hb and HHb with age (and of course also of tHb = O_2_Hb + HHb). This is expected since hemodynamic parameters are derived from the absorption coefficients by the Beer’s law assuming a linear relationship. Conversely, S_t_O_2_ remains almost the same, reflecting the physiological balance between O_2_Hb and HHb in the tissue.

### 4.3. Limitations of this study

The main limitations of this study are the reduced number of animals and the presence of different breeds. This last issue can be a bias, especially in dogs where different breeds have noticeably different anatomical characteristics. However, this is a pilot study mainly aimed at verifying the feasibility of TD NIRS measurements and not in providing normal (population) values. Therefore, the presented data cannot be generalized. Still, we believe that, as a reference, preliminary *in vivo* data should be preferred to *ex vivo* and *post mortem* data, as already discussed. Therefore, there is an area for improvement for future studies including a larger number of animals. Another limitation of this work is related to the use of a single source detector distance and of a homogeneous model for data fitting, despite the optically heterogeneity of the measured tissues. As a result, the presented data are average values over the sampled tissue volume (roughly 1 cm^3^ to an average depth of 1 cm from the skin surface). Indeed, the skin, including the epidermis, the dermal layer, the subcutaneous layer and the annexa (hair, glands) and the cutaneous, mimic, and superficial skeletal muscles vary with the species (61). Therefore, all the parameters measured here should be considered within the more general framework of species (and individual) differences. As a general rule, the skin of the horse is thicker than that of the sheep and dog (61). Thickness of the skin *per se* does not depend upon seasonality, but the quantity of subcutaneous fat, quality and abundance of hair do. Furthermore, blood supply to the skin and subcutaneous layers depends on external temperature and may also change in particular situations involving nervous regulation of the diameter of the peripheral arteries and veins. Finally, most horses have a dark skin, but that does not apply to many sheep and even canine breeds. All the factors mentioned here should be taken in account when discussing optical properties of the outer body layers of domestic species. In future, multi-distance measurements and heterogeneous model (coupled with anatomical *a priori* info on tissue structure) can help in obtaining more accurate values for the discrimination of different tissue type (e.g., fat, muscle). Furthermore, multi-wavelength NIRS measurements can provide more insight on other chromophores (e.g., water, collagen, lipid) for a complete tissue characterization. Finally, future studies should also discuss the participation of the color, length, and thickness of the hair (62).

### 4.4. Technical indications for NIRS use in veterinary field

The use and potential applications of the NIRS tool in veterinary medicine, including its use as a biomarker of skeletal muscle fatigue or in pathological conditions such as tissue ischemia, or during clinical procedures such as general anesthesia, are growing (63). Indeed, accurate estimate of S_t_O_2_ can be of paramount importance for example during surgical or diagnostic procedures especially in large animals (e.g., horse) where prolonged general anesthesia may lead to reduced oxygen uptake and altered gas exchange in the lungs that, combined with prolonged compression and immobility of skeletal muscle, can lead to impaired blood flow, tissue hypoxia and subsequent myopathies (21, 22, 64–66). Like in human applications, precise assessment of S_t_O_2_ can be also of vital impact when monitoring cerebral functions (e.g., in dog, cat, or rabbit) intraoperatively during anesthesia (67). Similarly, this tool has also helped to investigate cortical hemodynamics and functions after motor, cognitive, and somatosensory stimuli in dogs and sheep (28–33). Independently of the specific clinical or experimental application in the veterinary field, NIRS users should be aware of general technical aspects. Despite being compact and easy to use, CW NIRS commercial devices are strongly dependent of coupling with tissue and suffer from movement artifacts. Moreover, they provide relative hemodynamic changes with respect to a baseline. Quantitative values can be obtained only if DPF or (μ_s_’) is known and under the assumption of homogeneous medium. Differences in DPF among tissue types and animal species may exist as shown in this preliminary work, as well as heterogeneous structure can be present (e.g., fat covering muscle, scalp, skull covering brain). Optical properties and source-detector distance should be tailored to the specific tissue target. Moreover, CW NIRS devices have been designed for human applications and may not be optimal for use in veterinary field. Penetration depth of CW NIRS is limited, and it depends on source detector distance and strongly modulated by the optical properties. Most CW NIRS devices use a maximum distance of 4 cm that yields a maximum penetration depth of <2 cm. This prevents reaching deeper structure (e.g., brain cortex in horse, other internal organ). TD NIRS device can provide optical properties and absolute (i.e., not relative change) hemodynamic parameters of the measured tissue being less affected by movement artifacts and intrinsically calibrated (i.e., do not depend on literature data for μ_s_’ or DPF). However, they still have limited portability. For example, the NIRSBOX device used in this study has the dimension of a shoe box with a reduced weight (<3 kg) and can be easily carried by an operator close to the animal, but it cannot be properly worn by the animal for free movement study. Indeed, the combination of TD NIRS (robust, quantitative device for assessment of baseline optical properties and DPF) and CW NIRS (wearable, lightweight devices for assessment of changes with respect to the baseline) can be an optimal solution for animal studies.

## 5. Conclusion

In this work we provided preliminary *in vivo* measurement of the tissue optical properties (μ_a_, μ_s_’, and DPF) and of the hemodynamic parameters (O_2_Hb, HHb, tHb and S_t_O_2_) of domestic animals. Specifically, we focused on the skeletal muscle in dog and horse and on the head of dog and sheep. These parameters are obtained by means of state-of-the-art time domain (TD) NIRS systems and by applying physical model for photon diffusion. The results suggests that TD NIRS *in vivo* measurements on domestic animals are feasible, and that significant differences in the optical and hemodynamic properties among tissue types and species exist. Future work will increase the number of animals and improve the physical modeling to also consider heterogeneity in tissue type. Dedicated NIRS device should be used for optimized measurements in the veterinary field.

## Data availability statement

The raw data supporting the conclusions of this article will be made available by the authors, without undue reservation.

## Ethics statement

The animal studies were approved by Italian Health Ministry according to Directive 2010/63/EU (n. 457/2016-PR, 919/2017-PR; n. 0022387/2020). The studies were conducted in accordance with the local legislation and institutional requirements. Written informed consent was not obtained from the owners for the participation of their animals in this study because verbal informed consent was gained from each horse and dog owner prior to taking part in this research, written consent was deemed unnecessary as no personal details of the participants were recorded.

## Author contributions

BC, ED, MM, AT, LS, MC, and GV contributed to conception and design of the study. DoZ, DaZ, VR, GG, PS, LP, MM, and ED organized and performed the measurement on horses. ER, MC, GV, MM, and ED organized and performed the measurement on sheep. ED organized and performed the measurement on dogs. AT, LF, LS, DC, MB, and ML performed the measurements. LF, CA, LS, and LQ performed the data analysis. AT wrote the first draft of the manuscript. All authors contributed to the article and approved the submitted version.

## Funding

This work was partially supported by PNRR - Missione 4 “Istruzione e Ricerca” - Componente C2 Investimento 1.1 “Fondo per il Programma Nazionale di Ricerca e Progetti di Rilevante Interesse Nazionale (PRIN) 2022 (Grant Number 20227EPKW2).

## Conflict of interest

AT, DC, MB, and ML are cofounders of PIONIRS Srl.

The remaining authors declare that the research was conducted in the absence of any commercial or financial relationships that could be construed as a potential conflict of interest.

## Publisher’s note

All claims expressed in this article are solely those of the authors and do not necessarily represent those of their affiliated organizations, or those of the publisher, the editors and the reviewers. Any product that may be evaluated in this article, or claim that may be made by its manufacturer, is not guaranteed or endorsed by the publisher.
